# Removal of Tetracycline in Sewage and Dairy Products with High-Stable MOF

**DOI:** 10.3390/molecules25061312

**Published:** 2020-03-13

**Authors:** Kan Li, Jing-jing Li, Ni Zhao, Ying Ma, Bin Di

**Affiliations:** 1 Jiangsu Key Laboratory of Drug Design and Optimization, China Pharmaceutical University, Nanjing 210009, China; likan15@163.com (K.L.); 3219010178@stu.cpu.edu.cn (N.Z.); 2 Joint Laboratory on Key Technology of Narcotics Control, China Pharmaceutical University, Nanjing 210009, China; 3 National Institutes for Food and Drug Control, Beijing 100053, China

**Keywords:** MOF, adsorption and removal, tetracycline

## Abstract

Serious environmental and human health problems caused by the abuse of antibiotics have attracted worldwide concern. Recently, metal–organic frameworks (MOFs) with high porosity have drawn wide attention for their effects in the adsorption and removal of pollutants from complex matrices. Herein, a high-stable metal organic framework (MOF), i.e., ((ZnCl_2_)_3_(*L*)_2_·DMF)_n_, where *L*=1,3,5-tris((pyridin-4-ylthio)methyl)benzene), MOF **1,** was applied to adsorb and remove tetracycline from sewage and dairy products. The results showed that MOF **1** exhibited a strong performance in the adsorption of tetracycline. The effects of initial pH values, adsorbent dose, contact time and ionic strength of the adsorption performance of MOF **1** were investigated. The adsorption kinetics best fit the pseudo-second order model, and the adsorption isotherms matched the Langmuir adsorption model well. It was indicated that both chemical adsorption and physical adsorption play an important role in the adsorption process, and the adsorption of tetracycline was homogeneous and occurred on a monolayer on the surface of MOF **1**. Additionally, the stability of MOF **1** and the details of the adsorption mechanism were also investigated. Thus, this study provides a new candidate for the application of MOFs-based adsorbents in the removal of antibiotics from sewage and dairy products.

## 1. Introduction

**Figure 1:**
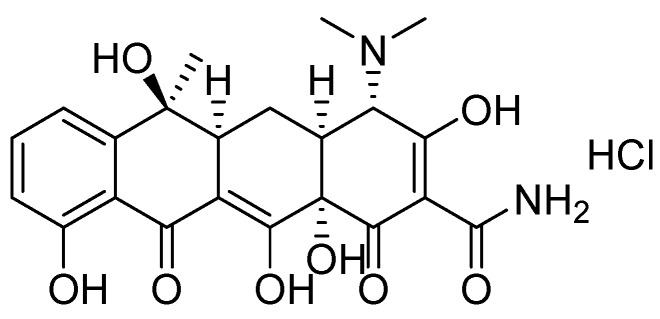
Structure of tetracycline hydrochloride.

Tetracycline hydrochloride (Figure 1) is one of the most common broad-spectrum tetracycline-based antibiotics, which is widely used for treating bacterial infections and as a feed additive in a variety of animal husbandry and aquacultures, due to its advantages such as its low cost, obvious effects, low toxicity and wide antimicrobial spectrum [1,2]. However, the abuse of antibiotics has become a major global issue since it causes serious environmental and food safety issues, due to their persistence and biological accumulation, and this abuse poses a latent threat to human health [3,4]. It was reported that the concentration of antibiotics in raw domestic sewage was in the range of 100 ng L^−1^–6 mg L^−1^, while the antibiotic concentration in pharmaceutical and hospital wastewater was detected in the range of 100–500 mg L^−1^ [5]. Meanwhile, the abuse of antibiotics also causes tetracycline residues in foods such as milk [6], meat [7], honey [8] and fish [9]. Excessive residual consumption or continual long-term intake with small doses of tetracycline can result in undesirable effects, such as gastrointestinal disturbance, anaphylactic reaction and hepatotoxicity, leading to a significant increase in antibiotic resistance of pathogenic microorganisms and a consequential increase in drug-resistant genes [10,11,12]. In the long term, the tetracycline will eventually enter the human body through the food chain and thus poses a serious threat to human health. Therefore, both the U.S. Food and Drug Administration (FDA) and the European Union (EU) have established the maximum residue limit (MRL) of tetracycline in milk as 300 ng mL^−1^ and 100 ng mL^−1^, respectively [10]. As a consequence, an economical and effective method was needed for the removal of tetracycline in water environments and foods.

To date, various different methods including adsorption [13], photochemical degradation [14], the biological method [15], membrane separation [16], chemical oxidation [17], electrochemical degradation [18] and advanced oxidation processes [19] have all been used to remove tetracycline from wastewater and milk. Among these methods, the removal of tetracycline with adsorbents has always been the most common method, due to its low operation cost, simple and practical operation, environmental friendliness, lack of secondary pollution and relatively high removal efficiency [20,21]. Recently, it was reported that many porous adsorbents have been successfully used to remove antibiotics from water and milk, including activated carbons [22], graphene oxide [23], nano-scaled zero valent iron [24] and multiwall carbon nanotubes [25]. However, many of these adsorbents generally show removal inefficiency and instability for antibiotic adsorption which limits their practical application [26]. Therefore, it is absolutely necessary to develop novel adsorbents with strong properties to remove tetracycline more effectively. In this regard, a method for removing tetracycline using a metal–organic framework (MOF) as a novel adsorbent has been proposed. MOFs have attracted widespread attention and achieved great development in the last two decades. MOFs are formed by self-assembly of metal cations or metal clusters with organic ligands (*L*) [27,28]. MOFs with large surface areas, structural diversity, high porosity and adjustable pore size or shape have demonstrated diverse potential applications in pollutant removal, energy storage, gas storage or separation, chemical sensing and catalysis [29]. Recently, the use of MOF materials as adsorbents has become one of the most widely used and promising applications. Therefore, it is necessary to develop other MOF materials with an excellent adsorption performance. 

In this study, based on our previous research [30], the performance of MOF **1** (i.e., ((ZnCl_2_)_3_(*L*)_2_·DMF)_n_, where *L*=1,3,5-tris((pyridin-4-ylthio)methyl)benzene) in the removal of tetracycline was investigated and the possible adsorption mechanisms were analyzed. The effects of the initial pH values, salt concentration, adsorbent dosage, contact time and the initial concentration of the adsorptive removal of tetracycline were evaluated. The strong adsorption performance of MOF **1** provides a new perspective for the removal of tetracycline.

## 2. Results and Discussion

### 2.1. Characterization

#### N_2_ Adsorption-Desorption Isotherms Analysis

**Figure 2:**
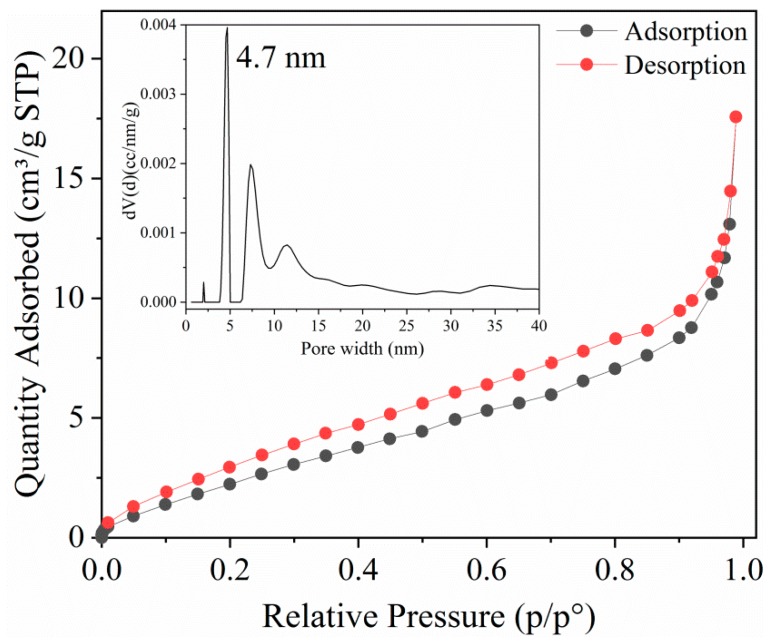
N_2_ adsorption-desorption isotherms of MOF **1**. (Inset is pore size distribution).

The adsorption parameters of the as-prepared adsorbents were obtained by the BET method and the result is illustrated in Figure 2. It can be seen from Figure 1 that MOF **1** had a type-H3 hysteresis and the curve of the adsorption–desorption isotherm was consistent with the typical type-IV isotherm [31], resulting from the volume filling theory of mesoporous. According to the BET and DFT methods, the specific surface area and pore width of MOF **1** were 11.86 and 4.7 nm, respectively.

### 2.2. Tetracycline Adsorption Studies

#### 2.2.1. Effect of pH on the Tetracycline Adsorption

**Figure 3:**
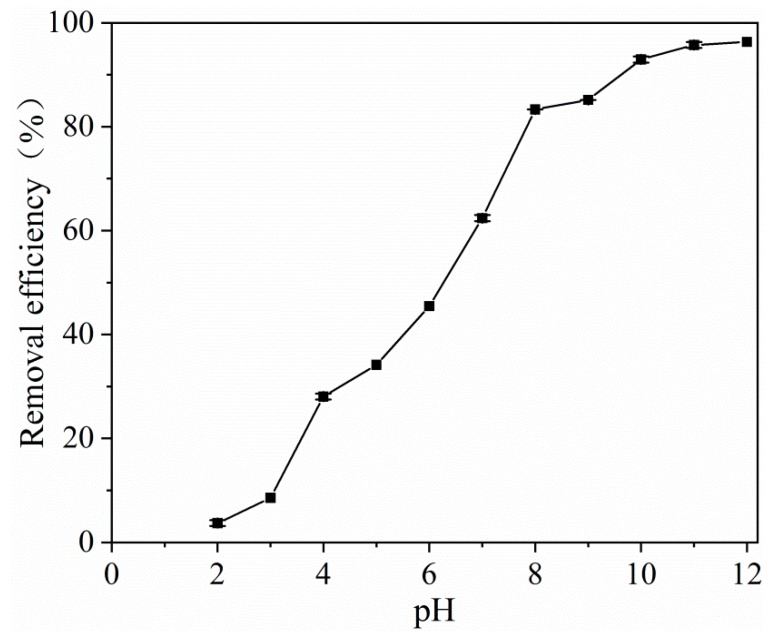
pH effect on the adsorption of tetracycline. (*C_0_* = 20 mg L^−1^, *V* = 10 mL, m (adsorbent) = 20 mg, T = 25 °C, t = 120 min).

The effect of pH (ranging from 2.0 to 12.0) on the adsorption of tetracycline on MOF **1** is shown in Figure 2. In Figure 3, it is indicated that the removal efficiency was very low in the highly acidic solution. With the increase in pH, the removal efficiency of tetracycline was improved, and then the efficiency remained unchanged when the pH > 10. This reason was that MOF **1** was unstable in the highly acidic environments, which led to the decomposition and loss of its adsorption performance. Accordingly, the optimum pH value of 10 was selected for the subsequent adsorption experiments.

#### 2.2.2. Effect of Ionic Strength on the Tetracycline Adsorption

**Figure 4:**
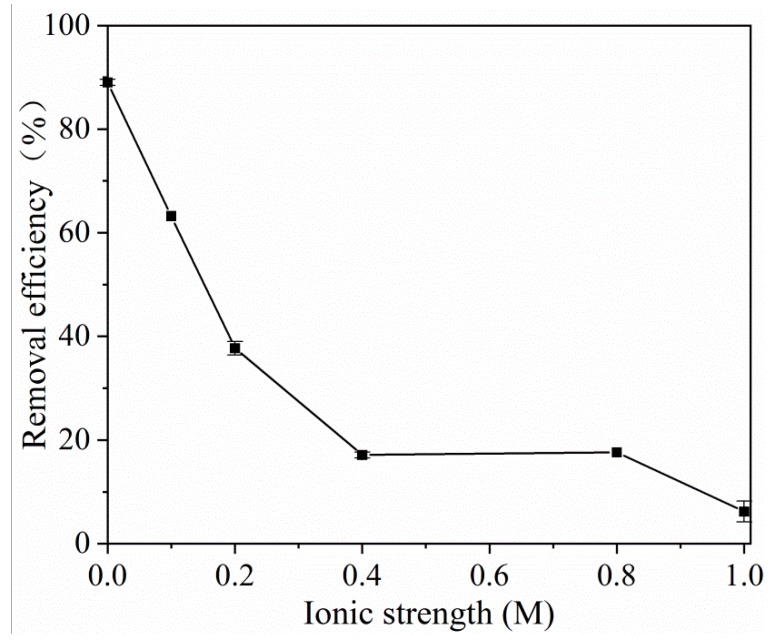
Ionic strength effect on the adsorption of tetracycline. (*C_0_* = 20 mg L^−1^, *V* = 10 mL, m (adsorbent) = 20 mg, T = 25 °C, t = 120 min).

The effect of ionic strength on the adsorption experiments was studied at pH = 10.0 and 25 °C in a 20 mg L^−1^ tetracycline solution containing 0–1.0 M NaCl. Figure 4 illustrates the removal efficiency of tetracycline versus the ionic strength. Obviously, NaCl has some negative impact on tetracycline adsorption. With the increase in the NaCl concentrations, the adsorption efficiency was decreased dramatically. It can be explained that the addition of ions might compete with tetracycline molecules for the adsorption sites on MOF **1**. In addition, the increased NaCl concentration might have caused the pores of the adsorbent to shrink, which brought about the failure of some adsorbed substances to enter the pores. Therefore, it can be concluded that coexisting ions have an adverse effect on the adsorption of tetracycline on MOF **1**.

#### 2.2.3. Effect of the Adsorption Dose on the Tetracycline Adsorption

**Figure 5:**
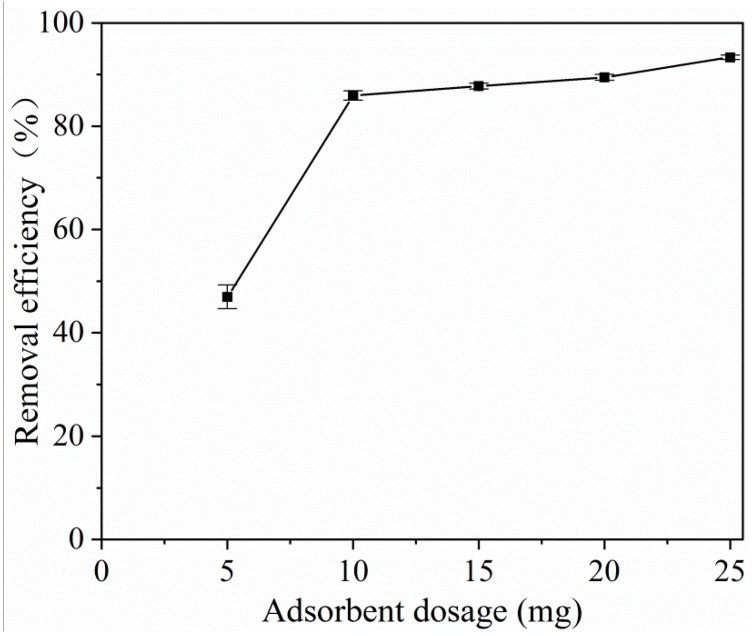
Adsorbent dosage effect on the adsorption of tetracycline. (*C_0_* = 20 mg L^−1^, *V* = 10 mL, T = 25 °C, t = 120 min).

Logically, at the same initial concentration of tetracycline, the increase in the amount of adsorbent will improve the removal efficiency. This is because more adsorbents provide more active sites for the adsorbate. Thus, we also examined the effect of the adsorption dosage on the tetracycline adsorption efficiency. The optimum adsorbent dose was determined using various amounts of MOF **1,** from 5 to 25 mg. Different amounts of MOF **1** were mixed with 20 mg L^−1^ tetracycline solution and shaken at 25 °C for 120 min to allow tetracycline to be sufficiently adsorbed. As shown in Figure 5, the optimum dose of MOF **1** was 20 mg and the removal efficiency could reach 90% when mixed with 20 mg L^−1^ tetracycline solution for 2 h.

#### 2.2.4. Adsorption Kinetics for Tetracycline on MOF 1

**Figure 6:**
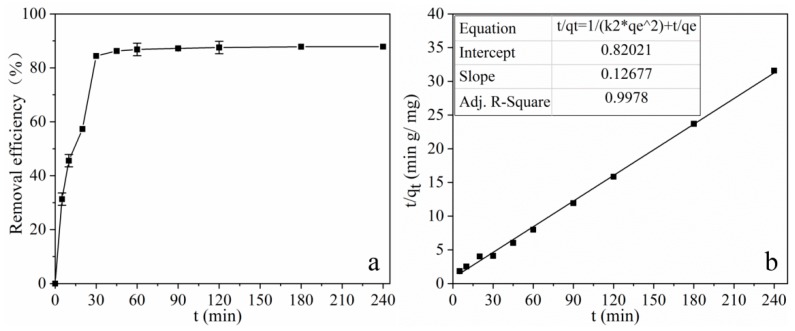
Effect of contact time on tetracycline removal (**a**) and pseudo-second-order kinetic plot (**b**) for the adsorption of tetracycline onto MOF **1**. (*C_0_* = 20 mg L^−1^, *V* = 10 mL, m (adsorbent) = 20 mg, T = 25 °C).

To evaluate the kinetic mechanism that controls the adsorption process of tetracycline, contact time experiments of a mixture of MOF **1** with tetracycline were carried out using the initial tetracycline concentration of 20 mg L^−1^ at pH 10.0 and shaken at 25 °C for a predetermined time (set as 5, 10, 20, 30, 45, 60, 90, 120, 180, 240 min). The effect of different contact times on removal efficiency is shown in Figure 6a. It can be concluded that tetracycline could be rapidly adsorbed by MOF **1** in 30 min, and the adsorption equilibrium could be reached in 120 min. Kinetic models were implemented to further elucidate and understand the mechanism of the effect of MOF **1** on solutes over time. Two kinetics equations, the pseudo-first-order and pseudo-second-order models, were used to fit the experiment data, respectively. The equations are as follows [32]:

(1) $$\ln\left(q_{e}-q_{t}\right)=\ln q_{e}-k_{1}t$$

(2) $$\frac{t}{q_{t}}=\frac{1}{k_{2}q_{e}^{2}}+\frac{t}{q_{e}}$$

where *q_e_* (mg g^−1^) is the adsorption capacity at equilibrium; *q_t_* (mg g^−1^) is the adsorption capacity of MOF **1** at time *t* (min); *k*_1_ (min^−1^) is the rate constant of the pseudo-first order kinetic model and *k*_2_ (g (mg min)^−1^) is the rate constant of the pseudo-second order kinetic model.

Simultaneously, to evaluate the applicability of two kinetic models, the relative deviation (Δ*q_e_*%) was employed as follows:

(3) $$\Delta q_{e}=\frac{q_{e,exp}-q_{e,cal}}{q_{e,cal}}\times 100\%$$

where the *q_e,exp_* represents the experimental value, the *q_e,cal_* is the calculated value obtained from the kinetic model.

The regression coefficient (*R^2^*), Δ*q_e_* and other fits of the kinetic model parameters acquired from the two kinetic models are shown in Table 1. As shown in Figure 6b and Table 1, it is clear that the pseudo-second order model was more suitable to describe the adsorption process of tetracycline onto MOF **1**. Therefore, it can be concluded that both chemical adsorption and physical adsorption played an important role in the adsorption process of tetracycline. 

#### 2.2.5. Adsorption isotherms for tetracycline on MOF **1**

The adsorption capacity of MOF **1** was investigated at pH 10.0 with known tetracycline concentrations (from 5 to 1500 mg L^−1^). As shown in Table 2, after the treatment of tetracycline solution with MOF **1**, the highest adsorption capacity can reach 30 mg g^−1^. These values are higher than those reported previously [33,34,35,36,37], which indicates that MOF **1** has broad application prospects in the removal of tetracycline from water. To further investigate the mechanism of tetracycline adsorption, the adsorption isotherm was used to analyze the adsorption behavior of MOF **1**. It was obtained by means of drawing the distribution diagram of the tetracycline equilibrium concentration in a solid–liquid phase at a constant temperature. Adsorption isotherms can provide not only the actual information on the capacity of the adsorbent, but also a deeper understanding of the reaction mechanism based on the model which is the most suitable for the data. In order to analyze the equilibrium data, two typical equilibrium models, the Langmuir adsorption isotherm and the Freundlich adsorption isotherm, were applied. The Langmuir model describes a monolayer sorption onto a homogeneous surface, while the Freundlich isotherm is applicable to both monolayer (chemisorption) and multilayer adsorption (physisorption) and based on the assumption that the adsorbate adsorbs onto the heterogeneous surface of an adsorbent. The two most commonly used adsorption models were expressed as follows [32]:

(4) $$\frac{c_{e}}{q_{e}}=\frac{1}{q_{m}K_{L}}+\frac{c_{e}}{q_{m}}$$

(5) $$\ln q_{e}=\ln K_{F}+\frac{1}{n}\ln c_{e}$$

where *c_e_* (mg L^−1^) is the equilibrium concentration of tetracycline, *q_e_* (mg g^−1^) is the equilibrium adsorption capacity, *q_m_* is the maximum adsorption capacity of MOF **1**, *K_L_* (L mg^−1^) and *K_F_* (mg g^−1^) are the Langmuir adsorption and the Freundlich constants, respectively. The *n* is an empirical parameter. The value of 1/*n* is usually between 0 and 1 and the size of 1/*n* indicates the effect of concentration on adsorption strength.

**Figure 7:**
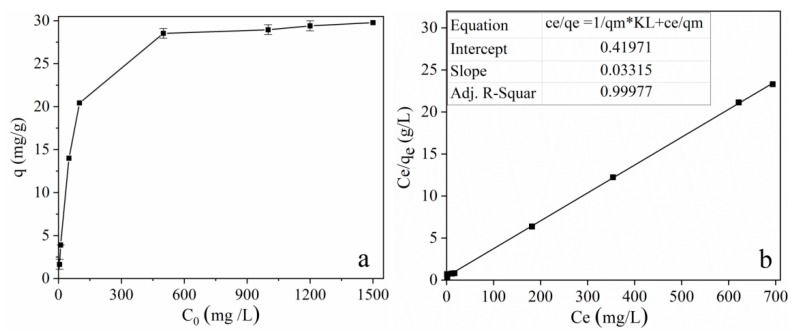
Sorption isotherm of tetracycline by MOF **1** (**a**) and adsorption isotherms fitted by the Langmuir models (**b**). (*V* = 10 mL, m (adsorbent) = 20 mg, T = 25 °C, t = 24 h).

The fitting results are represented in Figure 7b and Table 2. At 298 K, the goodness of fit, as indicated by the *R^2^* values, for tetracycline was 0.9998 and 0.8111 for the Langmuir equation and the Freundlich equation, respectively. Thus, from Table 2, the *R^2^* values for both models show that adsorption of the tetracycline by MOF **1** was in accordance with the Langmuir adsorption model. This indicates that the adsorption of tetracycline is homogeneous and occurs on a monolayer on the surface of MOF **1**, which is significant, and most likely the dominant mechanism. Moreover, the adsorption capacity of MOF **1**, calculated according to Equation (6), was 30.17 mg g^−1^. The calculation result is consistent with the experimental result (29.78 mg g^−1^). This again proves that the adsorption isotherm model is in accordance with the Langmuir model. At the same time, the material has a larger adsorption capacity than previously reported, indicating that MOF **1** has strong application prospects in the removal of tetracycline.

In order to compare the adsorption properties of the material reported in this study and other adsorption materials for tetracycline, we carried out the corresponding comparative analysis. The results are shown in Table 3. It can be seen from the comparative analysis in Table 3 that MOF **1** has certain advantages in its adsorption capacity.

#### 2.2.6. Potential Mechanism of Tetracycline Adsorption

According to the experimental results, the adsorption kinetics and isotherms data of tetracycline on MOF **1** indicated that the pseudo second-order kinetic and Langmuir models best described the adsorption process. We speculate that there are π–π interactions, electrostatic interactions and hydrogen bonding between tetracycline and MOF **1** [32,38,39]. This seems plausible since tetracycline contains multiple phenolic hydroxyl groups and plentiful conjugated benzene ring structures which can interact via hydrogen bonding and π–π interactions with the benzene ring and pyridine rings of MOF **1**. In addition, different pH values affected the adsorption behavior, indicating that there is still an electrostatic interaction between tetracycline and MOF **1**. 

### 2.3. Study on the Stability of MOF **1**

**Figure 8:**
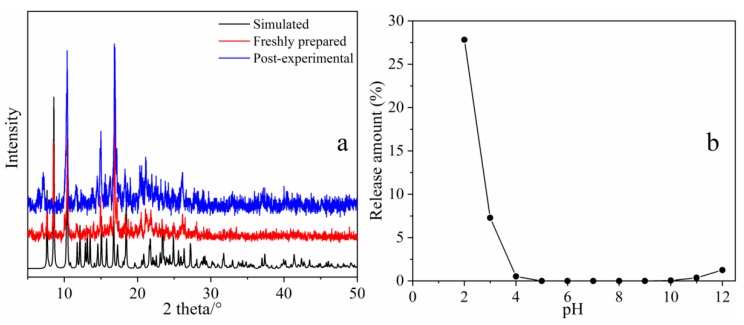
XRD spectra of MOF **1** (**a**) and the amount of Zn^2+^ released by MOF **1** in different pH solutions (**b**).

Considering that the stability of the adsorbent is one of the most important issues in practical applications, the stability of MOF **1** was further investigated. Before and after the adsorption experiment, PXRD was used to confirm whether the crystal structure of MOF **1** was intact. Meanwhile, in order to confirm the stability of the adsorbent, a degradation test of MOF **1** was conducted in water and aqueous solutions with different pH values. The amount of Zn^2+^ released from MOF **1** was quantitatively detected by Inductively Coupled Plasma Optical Emission Spectrometer ICP-OES after being placed in water for 7 days or shaken for 24 h in different pH solutions at room temperature. The XRD pattern of MOF **1** is shown in Figure 8a. It can be obtained from Figure 8a that the peak position and intensity of the spectrum before and after the adsorption experiment remain basically the same, indicating that the structure of MOF **1** remains intact after the adsorption experiment. In our previously reported studies, it was confirmed that MOF **1** is sufficiently stable in water, and the stability of adsorbents with pH values in the range of 2–9 has also been studied. In view of the better adsorption effect of this adsorbent on tetracycline in high pH solutions, we investigated the stability of MOF **1** in the pH range of 10–12. As shown in Figure 8b, in the solution of pH 10–12 the amount of Zn^2+^ released from MOF **1** was negligible (less than 1.3%). The result indicated that the adsorbent maintained sufficiently stability in the pH range of 4–12.

### 2.4. Treatment of Tetracycline in Sewage and Dairy Products

In order to verify the effectiveness of MOF **1** for the treatment of tetracycline in a real-world situation, practical experiments were conducted to remove tetracycline from already prepared wastewater and milk. Specifically, polluted water (10 mL) and milk (10 mL) containing 5 and 50 mg L^−1^ tetracycline were mixed with MOF **1** (20 mg) in a 20 mL glass centrifuge tube and shaken at 25 °C for 24 h. The max removal efficiency of tetracycline at the two wastewater concentrations by MOF **1** reached 96.3 and 79.6%, respectively (Table 4). While at the milk concentrations of 5 and 50 mg L^−1,^ the highest removal efficiency of tetracycline reached 99.2 and 71.2%, respectively (Table 4). These results show that MOF **1** can be practically used as an effective adsorbent for removing tetracycline from sewage and dairy products.

## 3. Experimental Section

### 3.1. Chemicals and Methods

Reagents including triethylamine (Et_3_N), sodium chloride (NaCl), diethyl ether, hydrochloric acid (HCl), sodium hydroxide (NaOH), *N*,*N*-Dimethylformamide (DMF) were obtained from Nanjing Chemical Reagent Co., Ltd (Nanjing, China). Zinc chloride (ZnCl_2_), 4-mercaptopyridine, 1,3,5-tris(bromomethyl)benzene and tetracycline hydrochloride were purchased from Aladdin (Aladdin Reagent (Shanghai, China) Co., Ltd.). Deionized water was prepared through a Miaozhiyi water purifier (Nanjing Miaozhiyi Electronic Technology Co., Ltd, Nanjing, China). All of the above chemicals were analytical reagent grade and used without purification.

The tetracycline adsorption and removal experiment was carried out on a SHIMADZU UV-1800 (Shimadzu Corporation, Kyoto, Japan). The N_2_ adsorption–desorption isotherms were performed on a Quantachrome Autosorb iQ (Quantachrome, Florida, FL, USA) analyzer and the adsorption parameters of MOF **1** were obtained by the Brunauer-Emmett-Teller (BET) method and the Density Functional Theory (DFT). The powder X-ray diffraction (PXRD) data were collected on a Bruker D8 Advance diffractometer (Bruker Corporation, Karlsruhe, Germany) with Cu Kα radiation (λ = 1.5406 Å) and 2θ ranging from 5° to 50° at room temperature. Quantitative detection of metal ions in solution with Inductively Coupled Plasma Optical Emission Spectrometer (ICP-OES) (Shimadzu Corporation, Kyoto, Japan).

### 3.2. Synthesis of Ligand (**L**) and Adsorbent (MOF **1**)

The *L* and MOF **1** were prepared according to our paper and reported previously [30]. Specifically, a mixture of 4-mercaptopyridine (15 mmol, 1.67 g), 1,3,5-tris(bromomethyl)benzene (5 mmol, 1.78 g) and Et_3_N (20 mmol, 2.0 g) in acetonitrile (30 mL) was stirred at 0 °C for 12 h, and then slowly warmed to room temperature. After the reaction was completed, the reaction solution was filtered to obtain a solid, and then washed with acetonitrile, deionized water, and ether three times. The residue was vacuum dried to obtain *L*. After obtaining the organic *L*, the acetonitrile solution (1.5 mL) of ZnCl_2_ (0.030 mmol) was layered on a DMF solution (1.5 mL) of the *L* (0.02 mmol) in a test tube. Then 1.5 mL of 1:1 acetonitrile/DMF buffer solution was layered between the top and bottom layers to allow slow diffusion for 3 days. Following this, MOF **1** was obtained.

### 3.3. Sample Preparation

The sewage samples were obtained from sewage treatment plants in Nanjing and Hangzhou (named NJ, HZ respectively). The solid insoluble particles were removed by filtration, and the filtered sample was stored in a 4 °C refrigerator.

The dairy products, including pure milk and yogurt, were purchased from a local supermarket and prepared as follows [40]: 2.0 mL dairy products was treated with 6.0 mL acetonitrile to precipitate protein and extract the analyte. Subsequently, the mixture was vortexed for 2 min and sonicated for 30 min, followed by centrifugation for 7 min at 6000 rpm. The milk also needed to be degreased with 4.0 mL normal hexane for three repetitions. The obtained extract was dissolved again with 10 mL of ultrapure water after filtration and evaporation.

### 3.4. Adsorption Experiments

The entirety of the adsorption experiments were performed in the sequence of batch adsorption. The 2000 mg L^−1^ stock solution of tetracycline was obtained by dissolving 2.0 g of tetracycline hydrochloride into deionized water. The different concentrations of tetracycline solution used in the adsorption experiments were prepared by diluting the stock solution. The different pH values were adjusted by NaOH (0.1 M) or HCl (0.1 M). An ultraviolet-visible spectrophotometer was used for the measurement of tetracycline concentrations at 359 nm. The calibration curves of tetracycline were measured at a concentration of 0.1–30 mg L^−1^. The adsorption capacity and removal efficiency of MOF **1** were calculated according to the following formula [32]:

(6) $$q_{t}=\frac{\left(C_{0}-C_{t}\right)V}{m}$$

(7) $$Removal\;efficiency\;\left(\%\right)=\frac{\left(C_{0}-C_{t}\right)}{C_{0}}\times 100\%$$

where *C_0_* (mg L^−1^) is the initial concentration of tetracycline and *C_t_* (mg L^−1^) is tetracycline concentration at time *t* (min) after treatment with MOF **1**. *V* (L) is the volume of the tetracycline solution and *m* (g) is the mass of adsorbent.

## 4. Conclusions

In summary, we have demonstrated that a water-stable MOF material, MOF **1**, could serve as an excellent adsorbent for the removal of antibiotic tetracycline in sewage and milk. Adsorption kinetic studies indicated that the removal of tetracycline by MOF **1** was a pseudo-second-order process. Adsorption isotherms studies showed that the adsorption process fit the Langmuir model well, indicating that tetracycline adsorption is homogeneous and occurred on a monolayer on the surface of MOF **1**. In addition, MOF **1** exhibited a good adsorption capability toward tetracycline in sewage and dairy products, which can mainly be attributed to the strong interactions between MOF **1** and tetracycline through π–π interactions, hydrogen bonding and electrostatic interactions. Therefore, the adsorbent, MOF **1**, can be applied to the removal of tetracycline in polluted water and dairy products.

## Figures and Tables

**Table 1:** Kinetic parameters for adsorption of tetracycline.

	Pseudo-First-Order Kinetic				Pseudo-Second-Order Kinetic			
*q_e,exp_*	*k*_1_	*q_e,cal_*	*R* ^2^	Δ*q_e_*	*k*_2_	*q_e,cal_*	*R* ^2^	Δ*q_e_*
7.60	0.0403	2.59	0.8919	−67.13%	0.01959	7.89	0.9978	−3.65%

**Table 2:** Adsorption isotherm parameters for adsorption of tetracycline.

	Langmuir Adsorption Isotherm			Freundlich Adsorption Isotherm		
*q_m,exp_*	*q_m,cal_*	*K_F_*	*R* ^2^	*K_F_*	*n*	*R* ^2^
29.78	30.17	0.07898	0.9998	1.219	2.6483	0.8111

**Table 3:** Compare the adsorption process parameters of different absorbents to remove tetracycline from water.

Adsorbent	Adsorption Kinetics Model	Adsorption Isotherm Model	Maximum Adsorption Capacity (mg/g)	Reference
UiO-66	pseudo-second-order	Langmuir model	23.10	33
Nanocellulose	pseudo-second-order	Redlich–Peterson	7.73	34
Pumice stone	pseudo-second-order	Langmuir and Freundlich model	20.02	35
Chitosan	-	Langmuir model	23.92	36
Activated carbon	pseudo-second-order	Langmuir model	1.98	37
MOF 1	pseudo-second-order	Langmuir model	29.78	This study

**Table 4:** Results of removing tetracycline from sewage and dairy products with MOF **1.**

Samples	Sewage				Dairy Products			
Name	NJ		HZ		Pure milk		Yogurt	
Concentrations (mg L^−1^)	5.0	50.0	5.0	50.0	5.0	50.0	5.0	50.0
Removal efficiency (%)	96.3	79.7	90.6	76.0	97.3	63.5	99.2	71. 2
